# The associations of the number of medications and the use of anticholinergics with recovery from tubal feeding: a longitudinal hospital-based study

**DOI:** 10.1186/s12877-020-01778-3

**Published:** 2020-09-29

**Authors:** Keiji Takata, Kentaro Oniki, Yuki Tateyama, Hiroki Yasuda, Miu Yokota, Sae Yamauchi, Norio Sugawara, Norio Yasui-Furukori, Junji Saruwatari

**Affiliations:** 1 Division of Pharmacy, Sakurajyuji Hospital, 1-1-1 Miyuki-kibe, Minani-ku, Kumamoto, 861-4173 Japan; 2 Division of Pharmacology and Therapeutics, Graduate School of Pharmaceutical Sciences, Kumamoto University, 5-1 Oe-honmachi, Chuo-ku, Kumamoto, 862-0973 Japan; 3 Division of Nursing, Sakurajyuji Hospital, 1-1-1 Miyuki-kibe, Minani-ku, Kumamoto, 861-4173 Japan; 4 Division of Respiratory Medicine, Sakurajyuji Hospital, 1-1-1 Miyuki-kibe, Minani-ku, Kumamoto, 861-4173 Japan; 5 Division of Nutrition, Sakurajyuji Hospital, 1-1-1 Miyuki-kibe, Minani-ku, Kumamoto, 861-4173 Japan; 6 Department of Psychiatry, Dokkyo Medical University School of Medicine, 880 Kitakobayashi, Mibu, Shimotsuga, Tochigi, 321-0293 Japan; 7 Department of Clinical Epidemiology, Translational Medical Center, National Center of Neurology and Psychiatry, 4-1-1 Ogawa-Higashi, Kodaira, Tokyo, 187-8551 Japan

**Keywords:** Dysphagia, Multiple medicines, Polypharmacy, Anticholinergics, Tubal feeding

## Abstract

**Background:**

Several medications, such as anticholinergics, are considered to affect the swallowing function adversely; however, whether or not anticholinergics or polypharmacy should be avoided to prevent eating dysfunction in elderly populations remains unclear. We therefore examined whether or not the number of medications or the use of anticholinergics was associated with recovery from tubal feeding in elderly inpatients.

**Methods:**

We conducted a retrospective 1-year observation study in 95 Japanese hospitalized patients (83.3 ± 9.7 years old) receiving nutrition through a feeding tube. The anticholinergic cognitive burden scale (ACBs) was used as an index for quantifying the anticholinergic action.

**Results:**

Thirty-six (37.9%) subjects recovered from tubal to oral feeding during the observation period. The logistic regression models showed that an increased number of prescribed medications and an increase in ACBs decreased the incidence of recovery from tubal feeding (odds ratio [95% confidence interval]: 0.66 [0.50–0.87], *P* = 0.003 and 0.52 [0.29–0.92], *P* = 0.024, respectively). Furthermore, the cumulative incidence of recovery from tubal feeding was significantly lower in the subjects who were given an additional ≥3 medications during the observation period than in those who were not (hazard ratio [95% confidence interval]: 0.08 [0.01–0.59], *P* = 0.014).

**Conclusions:**

The findings of this study suggest that an increased exposure to medications, especially anticholinergics, may be an important factor interfering with recovery from tubal feeding in hospitalized elderly patients.

## Background

Dysphagia is a swallowing disorder that affects the oral, pharyngeal, and/or esophageal function due to anatomical or physiological dysfunction [1, 2]. Dysphagia is a major health problem, especially in the elderly. The prevalence of dysphagia has been reported to range from 10 to 27% among older community-dwelling residents and about 50% in nursing home residents [1]. Dysphagia is strongly related to aspiration pneumonia, a decreased dietary intake, malnutrition and dehydration, all of which are factors that reduce the activity of daily living (ADL) and quality of life (QOL) in the elderly [3–5].

Normal swallowing is divided into three major phases: the oral phase, the pharyngeal phase and the esophageal phase [6]. It is a rapid and well-coordinated sequence of almost simultaneous muscle activities [6]. The underlying pathophysiology of dysphagia is complex. Dysfunction in dopamine-related and non-dopamine-related pathways, changes in cortical networks related to swallowing and peripheral mechanisms have been implicated in the pathogenesis of dysphagia [1, 7–9]. Glutamate and its analogues are the primary neurotransmitters involved in the stimulation of the oropharyngeal swallow mechanism [8]. However, the supramedullary pathways may be also regulated by dopamine and acetylcholine [8, 9]. Subcortical structures, including the basal ganglia, amygdala and hypothalamus, may exert both facilitatory and inhibitory influences on swallowing via dopaminergic actions [1, 8, 10]. A decline in skeletal muscle mass and/or strength might be also associated with dysphagia [11]. Therefore, aging and many kinds of diseases, such as Parkinson’s disease, dementia and cerebrovascular disease, affect the central swallowing network, peripheral nerves and muscles and have thus been reported as major risk factors for dysphagia [1, 12, 13]. In addition, pharmacological treatments are also associated with the development or progression of dysphagia. It has also been reported that some medications, such as anticholinergics, may affect the swallowing function adversely in the elderly [4, 14].

The use of multiple medicines, generally referred as polypharmacy, is common in older population with multimorbidity in whom one or more medicines may be used to treat each condition [15]. Although the definitions of polypharmacy vary, polypharmacy is related to poor health outcomes, including adverse drug events, medication non-adherence and a worse QOL in elderly population [16, 17]. A number of commonly used prescribed medications inhibit the function of smooth and striated muscles through pharmacologic actions on the central nervous system, neuromuscular transmission or myotoxic effects, thereby reducing the swallowing activity, bolus transit and esophageal sphincter movement [18]. Polypharmacy is also reported to be associated with the risk of frailty and malnutrition, which are strongly related to dysphagia [19, 20]. A previous epidemiological study showed a fairly high prevalence of swallowing difficulties in polypharmacy patients [21, 22]. However, whether or not the increased number of medications itself causes and/or aggravates dysphagia remains unknown.

Swallowing dysfunction can be caused by adverse drug reactions through esophageal injuries or induction of dysphagia [4, 8, 10]. Acetylcholine is a central and peripherally acting neurotransmitter that is responsible for excitation at the neuromuscular junction [8]. Anticholinergic activity is a common pharmacological action of many drugs [23] and is closely related to one of the underlying mechanisms for drug-induced xerostomia [4, 10, 24]. Elderly individuals may become more vulnerable to side effects from anticholinergics due to aging-related changes (e.g. decrease in brain muscarinic receptor density, acetylcholine transmission, blood-brain barrier integrity and drug metabolism) [24, 25]. Xerostomia decreases the formation of bolus of food and the ability of the mouth to send food to the pharynx during swallowing [4, 24]. In addition, the use of anticholinergics in elderly individuals is associated with a decline in the cognitive functions, which impairs the ability to focus on chewing and the sensory aspect of swallowing, thus delaying oral passage [23]. Furthermore, anticholinergic action causes gastroesophageal reflux by relaxing the lower esophageal sphincter [4]. Therefore, the above-stated actions of anticholinergics may be associated with dysphagia, but at present, few clinical studies regarding the direct relationship between the use of anticholinergics and dysphagia have been conducted in elderly populations.

Given the above, the present exploratory study investigated the potential impact of the number of prescribed medications and the use of anticholinergics on the recovery from tubal feeding in elderly hospitalized patients in order to clarify the potential relationship between pharmacological treatments and dysphagia.

## Methods

### Subjects and study protocol

This was a single-center, retrospective observational study, including new elderly (> 60 years old) patients hospitalized at Sakurajyuji Hospital (Kumamoto, Japan), which is a hospital that accepts chronic-phase patients, especially elderly patients, but not those in the acute phase, from April 2014 to December 2016. A longitudinal analysis was conducted with an observation period of 1 year among all 95 patients who had dysphagia and received nutrition through a feeding tube during hospitalization. The presence of dysphagia was assessed using a food test that involved eating pudding or jelly [26, 27]. The primary outcome was the recovery from tubal to oral feeding. For patients whose score on both the food test and the modified water swallowing test [26, 28] was ≥4, a speech pathologist and nurse started training for oral feeding. The patients were then screened for whether or not they were able to be fed orally by a physician. The patients who recovered from tubal feeding were defined as those who orally consumed all three meals each day.

The study observation started from the day at which each patient started tubal feeding (i.e. baseline). The endpoint was set at the 365th day after starting the observation of patients who had been receiving nutrition through a feeding tube or at the day when the patients recovered from tubal feeding during the observation period. For patients who did not recover from tubal feeding but were discharged from the hospital during the observation period, the endpoint was set at the day of discharge.

### Clinical information

We retrospectively obtained demographic and clinical data at the baseline and endpoint from the patients’ medical records. During the observation period, the date of discharge (regardless of the reason) was also collected to calculate the length of hospitalization after starting tubal feeding in each subject. In the present study, the Barthel index (BI) [29] and Hasegawa dementia rating scale-revised (HDS-R) were used as indices of the ADL [30] and cognitive function [31], respectively. The consciousness level in the patients with cerebrovascular disease was assessed by the Japan Coma Scale (JCS), which is the most extensively adopted scale in Japan because of its simplicity and applicability [32, 33]. The number and type(s) of prescribed medication(s) in each patient during the observation period were collected and reviewed once after the endpoint. Therefore, we did not interfere with the decisions on the type and number of prescribed medications for this retrospective study. An anticholinergic cognitive burden scale (ACBs), which is an index for quantifying anticholinergic action [25], was used for the evaluation of the anticholinergic effect.

### Statistical analyses

Continuous and categorical variables are presented as the median (range) and number (%), respectively. We used the Mann-Whitney *U*-test for the comparisons of categorical variables because the Shapiro-Wilk test showed that the variables were not normally distributed. We used the chi-square test for the comparisons of categorical variables. We used a logistic regression analysis to examine the associations of recovery from tubal feeding at the endpoint with the changes in the number of medications and the ACBs during the observation period and calculated the multivariable-adjusted odds ratios (ORs) and 95% confidence intervals (95% CIs). We used the Kaplan–Meier method to plot the cumulative proportion of recovery from tubal feeding or discharge from hospital by the increased number of medications or an increase in ACBs during the observation period, and the cumulative proportion was compared using the log-rank test. We also calculated the hazard ratios (HRs) and 95% CIs for the cumulative incidence of recovery from tubal feeding or discharge from the hospital using a Cox proportional hazards model. To confirm the precision of the parameters of the above-stated regression, we performed a nonparametric bootstrap analysis using 1000 replicated datasets generated by random sampling [34]. We evaluated the predictive performance of change in the number of medications during the observation period for detecting a recovery from tubal feeding using receiver operating characteristic (ROC) curves with calculations of the area under the curve (AUC). The cut-off value for changes in the number of medications was determined as the point with the shortest distance from the left upper corner of the graph. We used structural equation modeling [35] to assess the indirect effects of the changes in the number of medications and the ACBs on the recovery from tubal feeding at the endpoint. We adopted the following criteria for the goodness of fit on the structural equation modeling: root mean square error of approximation < 0.05, goodness of fit index (GFI) > 0.90, and adjusted GFI > 0.90.

We considered *P* < 0.05 to indicate statistical significance. We used Bonferroni’s method to correct multiple comparisons, and, after correcting for the number of comparisons made, we considered the values of *P* < 0.05/n to be statistically significant. The SPSS software package (version 23.0; IBM Japan Inc., Tokyo, Japan) was used for these statistical analyses.

## Results

The clinical characteristics of the study subjects at baseline are shown in Table 1. The total number of subjects receiving each drug category at baseline was also shown in Additional file 1. Thirty-six (37.9%) subjects recovered from tubal to oral feeding during the observation period. Thirty-one (32.6%) subjects did not recover but were discharged from the hospital within the 1-year observation period. The BI ranged from 0 to 20 in 96.8% of the subjects, so most of the subjects had a high need for nursing care. Since the HDS-R ranged from 0 to 10 in 88.4% of the patients, most subjects had dementia. In patients with cerebrovascular disease, although the values of the JSC ranged from 1 to 100 in patients with cerebrovascular disease at the time of admission, the median value was 10, implying that the underlying diseases were stable in most of the patients included in the current study. The clinical features at the baseline did not differ markedly between the patients who recovered from tubal feeding during the observation period (recovery group) and those who did not (non-recovery group), except for in age and the HDS-R (Table 1).

**Table 1:** Characteristics of the subjects at the baseline

	All subjects	Recovery group	Non-recovery group	*P* value
	(*N* = 95)	(*N* = 36)	(*N* = 59)	(recovery vs. non-recovery groups)
Sex (men/women)	34/61	13/23	21/38	1.000
Age (years)	84 (61–98)	81.5 (62–96)	86 (61–98)	0.069
Number of prescribed medications	5 (0–16)	5 (0–14)	6 (0–16)	0.401
ACBs	1 (0–6)	0 (0–6)	1 (0–4)	0.654
Route of tubal feeding (nasal/gastrostomy)	62/33	26/10	36/23	0.375
Cerebrovascular disease	71 (74.7%)	28 (77.8%)	43 (72.9%)	0.635
Wasting syndrome	39 (41.1%)	16 (44.4%)	23 (39.0%)	0.669
Parkinson’s disease	10 (10.5%)	2 (5.6%)	8 (13.6%)	0.310
Barthel index				
0–20	92 (96.8%)	34 (94.4%)	58 (98.3%)	0.141
21–40	1 (1.1%)	0 (0%)	1 (1.7%)	
> 40	2 (2.1%)	2 (5.6%)	0 (0%)	
HDS-R				
0–10	84 (88.4%)	28 (77.8%)	56 (94.9%)	0.068
11–20	7 (7.4%)	6 (16.7%)	1 (1.7%)	
> 20	4 (4.2%)	2 (5.6%)	2 (3.4%)	

The data are presented as the median [range] or number (%)

*ACBs* Anticholinergic cognitive burden scale, *HDS-R* Hasegawa dementia rating scale-revised

The recovery group represents the patients who recovered from tubal to oral feeding for all three meals per day during the observation period

**Fig. 1:**
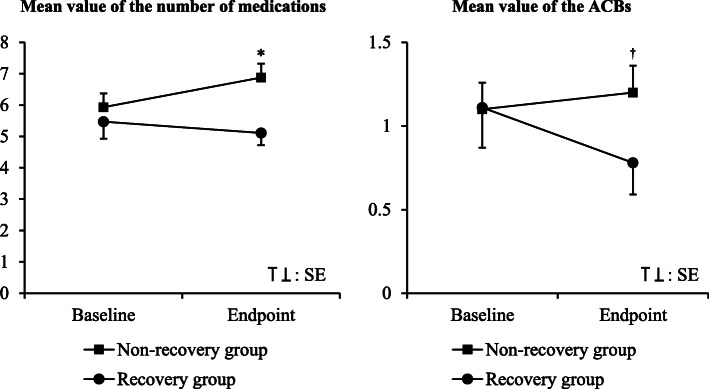
The changes in the number of prescribed medications and the ACBs. The *P* values were calculated by the Mann-Whitney U test. *P = 0.011, ^†^*P* = 0.035. ACBs, Anticholinergic cognitive burden scale; SE, standard error

Figure 1 shows the changes in the number of medications and the ACBs during the observation period. At baseline, there was no significant difference in the number of medications or ACBs between the recovery and non-recovery groups (Fig. 1, Table 1). However, at the endpoint, the number of medications and the ACBs were significantly higher in the non-recovery group than in the recovery group (*P* = 0.011 and *P* = 0.035, respectively) (Fig. 1). Furthermore, the results of logistic regression models showed that the increased number of prescribed medications or an increase in the ACBs during the observation period reduced the incidence of recovery from tubal feeding, regardless of the number of prescribed medications or ACBs at baseline (Table 2). To reduce the confounding effects by comorbidities, we tried to validate the findings using the same statistical procedures as described above separately in the subjects with cerebrovascular disease, Parkinson’s disease or wasting syndrome and those without (see Additional file 2). Accordingly, the results from these subgroup analyses indicated a similar tendency to those observed in all subjects, although the number of subjects with Parkinson’s disease was too small to analyze the association between pharmacotherapy and recovery from tubal feeding.

**Table 2:** The associations of recovery to oral feeding with prescribed medications or ACBs

	Recovery to oral feeding			
	Logistic regression analysis		Bootstrap analysis	
	Adjusted OR (95% CI)	*P* value	95% CI	*P* value
Model 1:				
The number of prescribed medications at the endpoint minus that at the baseline	0.66 (0.50–0.87) ^a^	0.003	0.41–0.84	0.002
Model 2:				
The ACBs at the endpoint minus that at the baseline	0.52 (0.29–0.92) ^b^	0.024	0.18–0.97	0.033

*OR* Odds ratio, *CI* Confidence interval, *ACBs* Anticholinergic cognitive burden scale, *HDS-R* Hasegawa dementia rating scale-revised

^a^ Adjusted for age, sex, gastrostomy tube, cerebrovascular disease, wasting syndrome, Parkinson’s disease, HDS-R and the number of prescribed medications at the baseline

^b^ Adjusted for age, sex, gastrostomy tube, cerebrovascular disease, wasting syndrome, Parkinson’s disease, HDS-R, the number of prescribed medications at the baseline and the ACBs at the baseline

**Fig. 2:**
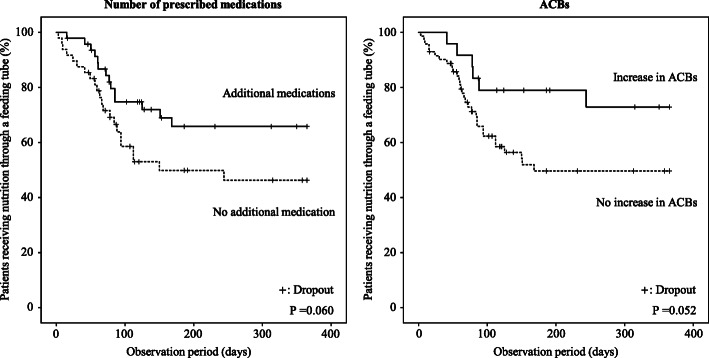
The prevalence of tubal feeding and prescribed medications or ACBs. The Kaplan-Meier curves indicate the incidence of recovery to oral feeding according to the changes in the number of medications or the ACBs. *P* values were calculated by the log-rank test. ACBs, Anticholinergic cognitive burden scale

**Fig. 3:**
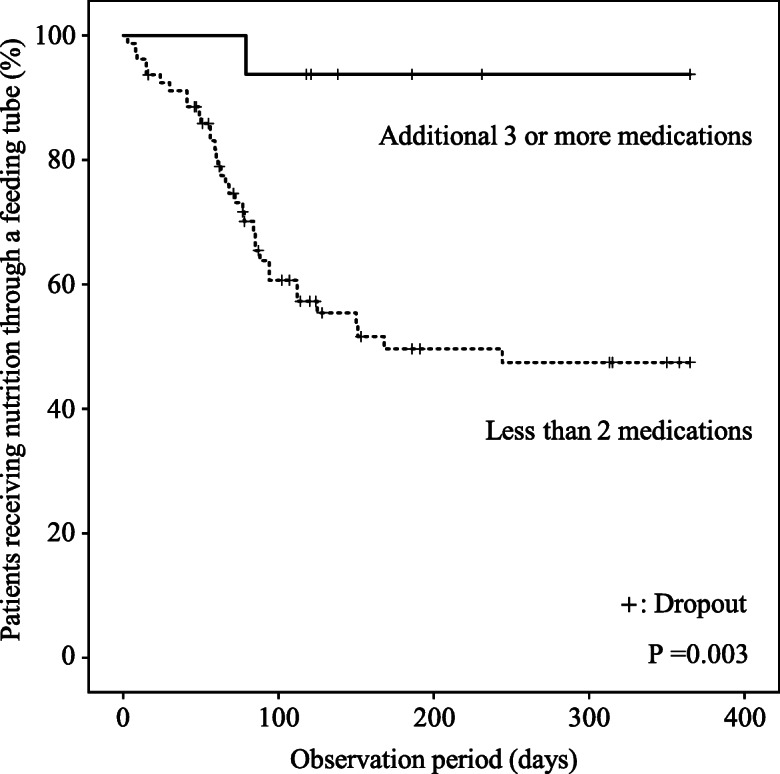
The prevalence of tubal feeding and an additional ≥3 medications. The Kaplan-Meier curve indicates the incidence of recovery from tubal to oral feeding in the subjects who were given an additional ≥3 medications during the observation period and in those who were not. *P* values were calculated by log-rank test

**Fig. 4:**
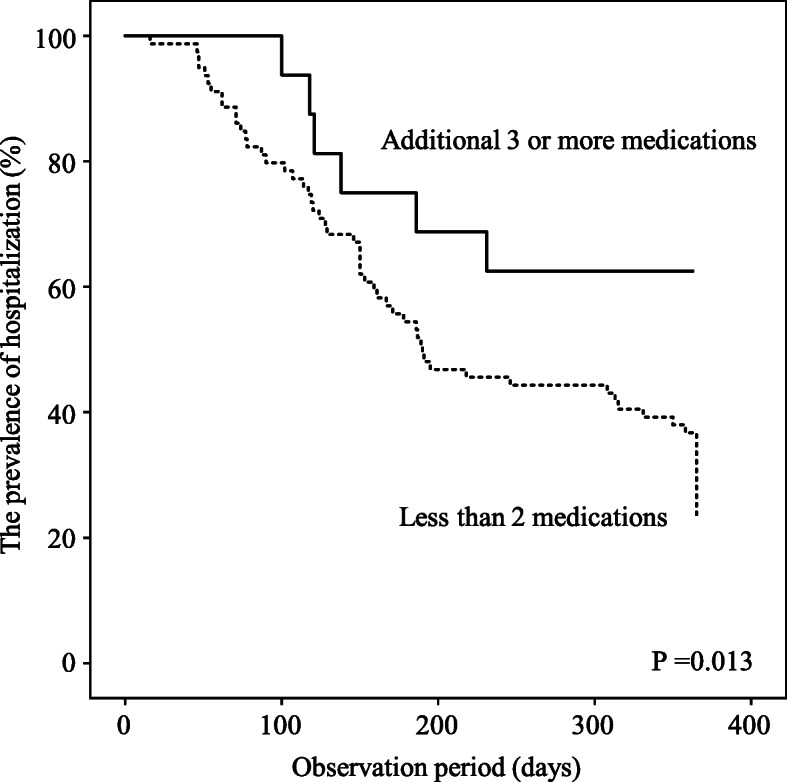
The prevalence of hospitalization and an additional ≥3 medications. The Kaplan-Meier curve indicates the incidence of discharge from hospital in the subjects who were given an additional ≥3 medications during the observation period and in those who were not. *P* values were calculated by log-rank test

To assess the effects of an increased number of prescribed medications or an increase in the ACBs on the recovery from tubal feeding during hospitalization, we estimated the incidence of recovery from tubal to oral feeding using a Kaplan–Meier curve (Fig. 2). During the observation period, the cumulative incidence of recovery from tubal feeding tended to be lower in the subjects who had an increased number of prescribed medications during the observation period than in those who did not (Fig. 2), but the difference did not reach statistical significance in the Cox regression hazard model (Table 3). The cumulative incidence of recovery from tubal feeding also tended to be lower in the subjects with an increased ACBs during the observation period than in those without an increased ACBs (Fig. 2 and Table 3). According to the ROC curve analysis, the change in the number of prescribed medications during the observation period was found to be a moderate predictor of recovery from tubal feeding (AUC 0.636, *P* = 0.027), and the optimal cut-off value was an additional ≥3 medications during the observation period (sensitivity, 25.4%; specificity, 97.2%) (see Additional file 3). The cumulative incidence of recovery from tubal feeding was significantly lower in the subjects who were given an additional ≥3 medications during the observation period than in those who were not (Fig. 3 and Table 3). To explore the clinical relevance of the association between an additional ≥3 medications and recovery from tubal feeding, we estimated the incidence of discharge from hospital during the observation period using a Kaplan–Meier curve (Fig. 4). The cumulative incidence of discharge was also significantly lower in the subjects who were given an additional ≥3 medications than in those with fewer (Fig. 4 and Table 4).

**Table 3:** The incidence of recovery to oral feeding and prescribed medications or ACBs

	Incidence of recovery to oral feeding			
	Cox regression analysis		Bootstrap analysis	
	Adjusted HR (95% CI)	*P* value	95% CI	*P* value
Model 1:				
No adding prescribed medication	1			
Adding prescribed medication (s)	0.65 (0.31–1.39) ^a^	0.269	0.23–1.65	0.333
Model 2:				
No adding 3 or more prescribed medication	1			
Adding 3 or more prescribed medications	0.08 (0.01–0.59) ^a^	0.014	0.03–0.42	0.024
Model 3:				
No increase in ACBs	1			
Increase in ACBs	0.36 (0.14–0.93) ^b^	0.035	0.07–1.14	0.083

*HR*, hazard ratio, *CI* Confidence interval, *ACBs* Anticholinergic cognitive burden scale, *HDS-R* Hasegawa dementia rating scale-revised

^a^ Adjusted for age, sex, gastrostomy tube, cerebrovascular disease, wasting syndrome, Parkinson’s disease, HDS-R and the number of prescribed medications at the baseline

^b^ Adjusted for age, sex, gastrostomy tube, cerebrovascular disease, wasting syndrome, Parkinson’s disease, HDS-R, the number of prescribed medications at the baseline and the ACBs at the baseline

**Table 4:** The incidence of discharge from the hospital and adding ≥3 prescribed medications

	Incidence of recovery to oral feeding			
	Cox regression analysis		Bootstrap analysis	
	Adjusted HR (95% CI)	*P* value	95% CI	*P* value
No adding 3 or more prescribed medication	1			
Adding 3 or more prescribed medications	0.33 (0.14–0.80) ^a^	0.014	0.07–0.92	0.032

*HR* Hazard ratio, *CI* Confidence interval, *ACBs* Anticholinergic cognitive burden scale, *HDS-R* Hasegawa dementia rating scale-revised

^a^ Adjusted for age, sex, gastrostomy tube, cerebrovascular disease, wasting syndrome, Parkinson’s disease, HDS-R and the number of prescribed medications at the baseline

**Fig. 5:**
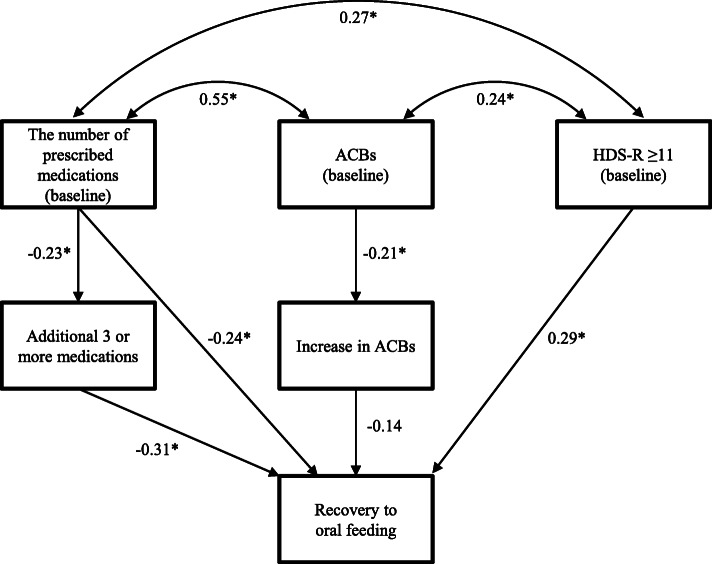
The structural equation modeling diagram regarding the recovery to oral feeding. Lines with numbers indicate significant paths with standardized partial regression (β) coefficients (*P < 0.05). Arrows indicate an association between two factors. The β values ranged from − 1 to 1, with a positive value representing a positive correlation and a negative value representing a negative correlation. ACBs, anticholinergic cognitive burden scale; HDS-R, Hasegawa dementia rating scale-revised

Furthermore, to verify the observed associations of an additional ≥3 medications (or increased ACBs) and recovery from tubal feeding in more detail, we performed a pathway analysis using structural equation modeling regarding the possible associations among medications, recovery to oral feeding, cognitive decline and neuromuscular dysfunction (Fig. 5). As the results, an additional ≥3 medications was significantly incorporated into the model and modeled directly and negatively on the incidence of the recovery to oral feeding. The association of an additional ≥3 medications with the recovery was more pronounced than that of the increased ACBs. Furthermore, the number of medications and ACBs at baseline was significantly associated with the HDS-R score at baseline, which was modeled directly on the recovery from tubal feeding. The structural equation model incorporating other potential risk factors (e.g. cerebrovascular diseases, Parkinson’s disease) for an impaired swallowing function could not be developed, as the parameters (e.g. standardized partial regression coefficients) did not converge.

The bootstrap analyses of logistic and Cox hazard regression models replicated the above-mentioned findings in 1000 datasets generated by random sampling of the original dataset (Tables 2, 3 and 4), indicating that the model we developed described the data adequately.

## Discussion

This exploratory study showed for the first time a potential effect of changes in the number of prescribed medications or the ACBs approximately 1 year after tubal feeding on the recovery to oral feeding in a hospitalized elderly population. The results of the present study suggest that increased exposure to medications, especially anticholinergics, may be associated with a reduced recovery from dysphagia in an elderly population.

A number of commonly used medications are often associated with the risk of dysphagia by decreasing the swallowing ability and reducing the esophageal sphincter movement [18]. The influence of medications on the development of dysphagia might be additive or interactively enhanced by the increased number of medications [36]. The results of this study showed that the changes in the number of medications and in the ACBs during hospitalization were potential important factors affecting the recovery from tubal feeding (Figs. 1 and 2, Tables 2 and 3, Additional file 2), while the number of medications and the ACBs at the beginning of tubal feeding did not affect the recovery within 1 year (Fig. 1). In particular, an additional ≥3 medications during hospitalization were strongly associated with disturbance of recovery from tubal to oral feeding (Fig. 3 and Table 3) as well as with the longer duration of hospital stays after tubal feeding (Fig. 4 and Table 4). Therefore, careful attention should be paid when adding medications to elderly patients on tubal feeding during hospitalization.

In the present study, we focused on the association between the anticholinergic action and dysphagia. Side effects of anticholinergics include various symptoms of the peripheral and central nervous systems (e.g. constipation, dry mouth, dry eye, tachycardia, urinary retention, shaking, confusion, delirium, falls, hallucinations and the cognitive function decline) [23, 24]. The anticholinergic effect impairs the swallowing ability by weakening the parasympathetic action transmitted by the muscarinic acetylcholine receptor [37]. Drying of the mouth is associated with dysphagia due to the reduced formation of a food bolus and the decreased ability of the mouth to send this bolus to the pharynx during swallowing [24]. A previous meta-analysis reported that anticholinergic effects were associated with a cognitive decline and an increased risk of falls and mortality [23]. Moreover, Richardson et al. suggested that the use of anticholinergics is strongly associated with the development of dementia [38], and Sura et al. indicated that one out of five elderly patients with dementia are prescribed with anticholinergics [39]. Furthermore, decline in cognitive function decreases the abilities of feeding and swallowing due to a decrease in cognition of food and lack of oral movement associated with food intake [40]. Indeed, in this study, the prevalence of low HDS-R (i.e. ≤10) at baseline was significantly greater in the non-recovery group that in the recovery group (Table 1). However, the present study showed that the recovery to oral feeding was associated with the ACBs, even after adjusting for the HDS-R at baseline (Tables 2 and 3). Nevertheless, while the detailed mechanisms underlying the association between anticholinergics and dysphagia remain unclear, the present findings may suggest that avoiding the use of anticholinergics might help prevent and treat dysphagia.

Dysphagia is a common co-morbidity of several diseases, such as dementia, Parkinson’s disease and cerebrovascular diseases, that directly or indirectly impact the swallowing process or swallowing response [1, 10]. However, multimorbidity, commonly defined as the co-existence of two or more chronic health conditions, is common in older populations [15]. In addition, there is a close relationship between an increased number of medications and the deterioration of the overall condition [16, 17]. Therefore, it is possible that the number of prescribed medications was a proxy for other factors, such as a decline in the general health. The findings from our subgroup analyses in the subjects with or without co-morbidities indicated the same tendency concerning the association between pharmacotherapy and recovery from tubal feeding as the results obtained in all subjects, although the numbers of subjects might not be sufficient to analyze the associations (Additional file 2). Furthermore, according to our structural equation modeling analysis, an additional ≥3 medications was significantly and directly associated with the reduced incidence of the recovery to oral feeding, but it was not influenced by the HDS-R score at baseline (Fig. 5). Therefore, these findings suggest that changes in the prescribed medications may be associated directly with a reduced recovery from tubal feeding. Nevertheless, further investigations considering the changes in not only the pharmacological treatments but also the overall condition of the hospitalized elderly patients are needed in order to verify the present results.

The subjects of the present study were newly hospitalized elderly patients receiving nutrition through a feeding tube. The subjects’ ADL and cognitive function were extremely low, and most had a high need for nursing care. Since the underlying causes of tubal feeding were serious diseases, such as cerebrovascular diseases, pneumonia and sarcopenia, most of the subjects likely had a high degree of frailty. Frail elderly individuals are reported to have reduced pharmacokinetics, especially concerning drug metabolic and excretion capabilities [41]. Another study showed a close relationship between polypharmacy and the occurrence of adverse drug events, and the relationship was more pronounced in patients with frailty than in those without frailty [20]. In recent years, the number of cases of falls caused by drugs has been high among frail elderly patients [42], so the concept of pharmacological frailty has been proposed [43]. Taken together, the present and previous findings suggest that it may be necessary to pay special attention to polypharmacy among elderly patients with frailty.

There are several limitations associated with the present study that should be noted. First, the present study retrospectively investigated a relatively small number of subjects in a single center. This design makes it difficult in some cases to differentiate between the effect produced by the medication itself and the medical condition, which can result in an under- or over-estimation of the effect of the studied medication on the swallowing function. The present findings thus warrant further interventional investigation evaluating the impact of the changes in the number of prescribed medications and ACBs on dysphagia in larger, more diverse populations. Second, the present study showed the possible relationship between polypharmacy and dysphagia, but we were unable to consider the effects of the individual types of drugs on dysphagia, except for anticholinergics. Third, although we performed regression analyses incorporating patient factors, such as age, sex, gastrostomy tube, cerebrovascular disease, wasting syndrome, Parkinson’s disease and HDS-R, as potential confounding factors (Tables 2, 3 and 4), we were unable to investigate the influence of other factors (e.g. the respiratory function, swallowing rehabilitation, oral care, nutritional status and dietary care) [4, 44] on the recovery from tubal nutrition. Finally, although the current study used a food test as well as the modified water swallowing test to evaluate whether or not the patients could start training for oral feeding, a fiberoptic endoscopic evaluation of swallowing and videofluoroscopic swallowing study are both considered to be the gold standard for evaluating individuals with oropharyngeal dysphagia [7, 26, 28]. Further large-scale studies incorporating other patient factors with more validated methods are therefore needed in order to verify the effects of pharmacological treatment on dysphagia.

## Conclusions

The results of this study suggest that an increased number of medications, especially anticholinergics, in hospitalized elderly patients receiving tubal feeding may be a risk factor hindering recovery to oral feeding. The present findings may be used to help improve the ADL and/or QOL in hospitalized elderly patients on tubal feeding, although further investigations are needed to verify and further clarify these findings.

## Supplementary information

**Additional file 1:.** Total number of study subjects receiving each drug category at baseline.

**Additional file 2:.** The associations of recovery to oral feeding with prescribed medications or ACBs separately in the subjects with cerebrovascular disease, Parkinson’s disease or wasting syndrome and those without.

**Additional file 3:.** The ROC curve of the change in the number of medications for recovery to oral feeding.

## Data Availability

The datasets analyzed during the present study are available from the corresponding author on reasonable request.

## Abbreviations

ACBs: Anticholinergic cognitive burden scale
ADL: Activity of daily living
AUC: Area under the curve
BI: Barthel index
CI: Confidence interval
GFI: Goodness of fit index
HDS-R: Hasegawa dementia rating scale-revised
HR: Hazard ratio
JCS: Japan Coma Scale
OR: Odds ratio
QOL: Quality of life
ROC: Receiver operating characteristic
