# Supplementary material for: The associations of the number of medications and the use of anticholinergics with recovery from tubal feeding: a longitudinal hospital-based study
Source: BMC Geriatr. 2020 Sep 29;20:373. doi: 10.1186/s12877-020-01778-3 (PMC7526129; doi:10.1186/s12877-020-01778-3)
Supplement: Supplementary file 1 — Additional file 1:. Total number of study subjects receiving each drug category at baseline. [file 12877_2020_1778_MOESM1_ESM.docx]

| **Additional file 1.** Total number of study subjects receiving each drug category at baseline. | | | |
| --- | --- | --- | --- |
|  | N |  | N |
| Ca channel blockers | 23 | Acetylcholinesterase inhibitors | 10 |
| Beta blockers | 17 | NMDA receptor inhibitors | 2 |
| ACE inhibitors | 13 | Alpha 1 blockers | 7 |
| ARBs | 16 | Muscarinic receptor antagonists | 3 |
| Vasodilators | 5 | DPP4 inhibitors | 7 |
| Warfarin | 13 | Sulfonylureas | 1 |
| DOACs | 4 | Alpha-glucosidase inhibitors | 1 |
| Antiplatelet drugs | 21 | Steroids | 6 |
| Antiarrhythmic drugs | 7 | NSAIDs | 0 |
| Diuretics | 31 | Acetaminophen | 4 |
| Statins | 9 | PPIs | 35 |
| XO inhibitors | 4 | Histamine-2 receptor antagonists | 16 |
| Other cardiovascular drugs | 1 | Gastric mucosal defense factor enhancing drugs | 14 |
| Typical antipsychotics | 8 | Osmotic laxatives | 20 |
| Atypical antipsychotics | 11 | Stimulant laxatives | 15 |
| SSRI | 4 | Intestinal drugs | 32 |
| Benzodiazepines | 16 | Other gastrointestinal drugs | 10 |
| Nonbenzodiazepines | 5 |  |  |
| Other hypnotics | 3 |  |  |
| Antiepileptics | 16 |  |  |
| Levodopa | 11 |  |  |
| Dopamine agonist | 6 |  |  |
| Other drugs for Parkinson’s disease | 12 |  |  |
| ACE, angiotensin converting enzyme; ARB, angiotensin II receptor blocker; DOAC, direct oral anticoagulants; XO, xanthine oxidase; NMDA, *n*-methyl-*d*-aspartate; DPP4, dipeptidyl peptidase 4; NSAIDs, non-steroidal anti- inflammatory drugs; PPI, proton pump inhibitor. | | | |
