# Supplementary material for: The associations of the number of medications and the use of anticholinergics with recovery from tubal feeding: a longitudinal hospital-based study
Source: BMC Geriatr. 2020 Sep 29;20:373. doi: 10.1186/s12877-020-01778-3 (PMC7526129; doi:10.1186/s12877-020-01778-3)
Supplement: Supplementary file 2 — Additional file 2:. The associations of recovery to oral feeding with prescribed medications or ACBs separately in the subjects with cerebrovascular disease, Parkinson’s disease or wasting syndrome and those without. [file 12877_2020_1778_MOESM2_ESM.docx]

| **Additional file 2.** The associations of recovery to oral feeding with prescribed medications or ACBs separately in the subjects with cerebrovascular disease, Parkinson’s disease or wasting syndrome and those without. | | |
| --- | --- | --- |
|  | Logistic regression analysis | |
|  | Adjusted OR (95% CI) | P value |
| **Patients with cerebrovascular disease**  Model 1:  The number of prescribed medications at the endpoint minus that at the baseline | 0.67 (0.49-0.92) ^a^ | 0.013 |
|  |  |  |
| Model 2:  The ACBs at the endpoint minus that at the baseline | 0.65 (0.34-1.23) ^b^ | 0.187 |
|  |  |  |
| **Patients without cerebrovascular disease**  Model 1:  The number of prescribed medications at the endpoint minus that at the baseline | 0.64 (0.34-1.22) ^a^ | 0.174 |
|  |  |  |
| Model 2:  The ACBs at the endpoint minus that at the baseline | 0.32 (0.07-1.42) ^b^ | 0.135 |
|  |  |  |
| **Patients with Parkinson’s disease**  Model 1:  The number of prescribed medications at the endpoint minus that at the baseline | Not calculated | Not calculated |
|  |  |  |
| Model 2:  The ACBs at the endpoint minus that at the baseline | Not calculated | Not calculated |
|  |  |  |
| **Patients without Parkinson’s disease**  Model 1:  The number of prescribed medications at the endpoint minus that at the baseline | 0.65 (0.49-0.87) ^a^ | 0.004 |
|  |  |  |
| Model 2:  The ACBs at the endpoint minus that at the baseline | 0.48 (0.25-0.92) ^b^ | 0.026 |
|  |  |  |
| **Patients with wasting syndrome**  Model 1:  The number of prescribed medications at the endpoint minus that at the baseline | 0.66 (0.41-1.05) ^a^ | 0.080 |
|  |  |  |
| Model 2:  The ACBs at the endpoint minus that at the baseline | 0.60 (0.25-1.43) ^b^ | 0.248 |
|  |  |  |
| **Patients without wasting syndrome**  Model 1:  The number of prescribed medications at the endpoint minus that at the baseline | 0.65 (0.45-0.92) ^a^ | 0.016 |
|  |  |  |
| Model 2:  The ACBs at the endpoint minus that at the baseline | 0.71 (0.32-1.57) ^b^ | 0.401 |
| ^a^ Adjusted for age, sex, gastrostomy tube, HDS-R and the number of prescribed medications at the baseline.  ^b^ Adjusted for age, sex, gastrostomy tube, HDS-R, the number of prescribed medications at the baseline and the ACBs at the baseline.  OR, odds ratio; CI, confidence interval; ACBs, anticholinergic cognitive burden scale; HDS-R, Hasegawa dementia rating scale-revised. | | |
