# Supplementary material for: The associations of the number of medications and the use of anticholinergics with recovery from tubal feeding: a longitudinal hospital-based study
Source: BMC Geriatr. 2020 Sep 29;20:373. doi: 10.1186/s12877-020-01778-3 (PMC7526129; doi:10.1186/s12877-020-01778-3)
Supplement: Supplementary file 3 — Additional file 3:. The ROC curve of the change in the number of medications for recovery to oral feeding. [file 12877_2020_1778_MOESM3_ESM.pptx]

## Slide 1
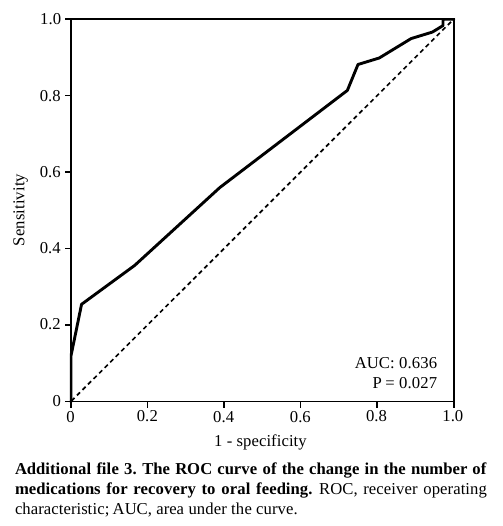

1.0
0.8
0.6
Sensitivity
0.4
0.2
AUC: 0.636
P = 0.027
0
1.0
0.8
0.2
0.4
0.6
0
1 - specificity
Additional file 3. The ROC curve of the change in the number of medications for recovery to oral feeding. ROC, receiver operating characteristic; AUC, area under the curve.
